# Electrocardiographic Early Repolarization in an Emergency Setting: The Subtleties of Electrocardiography

**DOI:** 10.7759/cureus.46253

**Published:** 2023-09-30

**Authors:** Gagan Neupane, Zed O Seedat, Touqir Zahra

**Affiliations:** 1 Internal Medicine, Florida Atlantic University Charles E. Schmidt College of Medicine, Boca Raton, USA; 2 Critical Care, Mercy Hospital St. Louis, Boca Raton, USA

**Keywords:** st-segment elevation, j-point elevation, end qrs notch, diffuse st elevation, early repolarization pattern

## Abstract

The electrocardiographic pattern of early repolarization (ER) is relatively common in the general population. In patients presenting to the emergency room with chest pain, it can be particularly challenging to distinguish ER from life-threatening subtle ST-segment elevation myocardial infarction (STEMI).

A 37-year-old male presented to the emergency department with sudden-onset, severe, non-radiating, central chest pain. The ECG showed Q waves in the inferior leads and a widespread end-QRS notch with J-point elevation mimicking ST elevation in the inferior and lateral precordial leads. Initial cardiac biomarkers were within normal limits. Serial cardiac biomarkers were unremarkable. Echocardiography showed no wall motion abnormalities. A review of prior records from a month ago revealed a similar presentation with similar ECG findings when he underwent cardiac catheterization, revealing normal coronary arteries. Since the ECG was unchanged from the prior one with negative cardiac biomarkers and a negative angiographic study a month ago, no further ischemic risk stratification was indicated.

Distinguishing ER from subtle STEMI in patients with acute chest pain can be challenging. A good clinical acumen, along with a comparison of prior ECGs, can aid in decision-making.

## Introduction

The pattern of early repolarization (ER) in ECG is common in cardiology practice, and the prevalence is noted to be variable between 2% and 31% [1]. Historically, the pattern of early repolarization has been regarded as a benign ECG variant and was first described by Shipley and Hallaran in 1936 [2]. However, recently, there has been an evolving concept of linkage between early repolarization patterns and sudden cardiac death [1,3-5]. There is considerable variation in the definition of early repolarization. In general, early repolarization is characterized by J-point elevation and QRS notching in multiple leads, especially in inferior and left precordial leads [1].

In patients presenting to the emergency department with chest pain, it can be particularly challenging to distinguish ER from life-threatening subtle ST-segment elevation myocardial infarction (STEMI) leading to invasive procedures or the administration of thrombolytic agents; Sharkey et al. [6] reported the administration of a thrombolytic agent in 11% of patients who did not have an actual STEMI. Sometimes, even with trained eyes, the distinction between subtle STEMI and ER may be difficult, especially in the acute setting of chest pain when cardiovascular risk factors indicate moderate to high risk. Therefore, cardiac risk stratification along with a clear consensus can help achieve good clinical acumen in emergencies. Here, we report the case of a young male with acute chest pain with ECG features of benign ER, focusing on risk stratification, overcalls, and at the same time avoiding undercalls while evaluating these types of patients and differentiating them from life-threatening subtle STEMI.

## Case presentation

**Figure 1:**
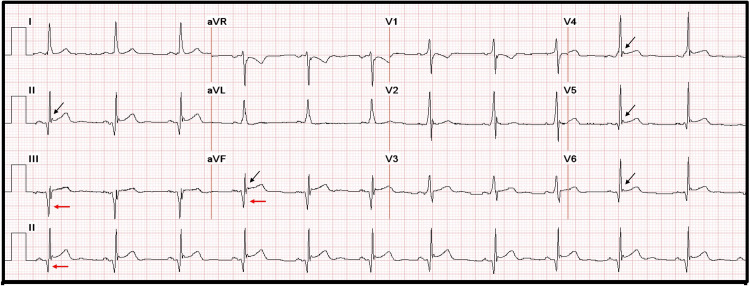
ECG showing an end-QRS notch or slur on the downslope of prominent R wave (black arrows), J point ≥ 0.1 mV in contiguous leads, and narrow QRS duration suggestive of benign ER Red arrows: Pathologic Q waves in leads II, III, and aVF ER: Early repolarization, aVF: Arteriovenous fistulas

A 29-year-old male with a history of smoking presented to the ED with sudden-onset, severe, non-radiating central chest pain of two hours duration. He also mentioned having occasional palpitations two to three times a week lasting for about half an hour for the last few years. The patient denied dyspnea, dizziness, nausea, vomiting, diaphoresis, fever, chills, and abdominal pain. On presentation, he was hemodynamically stable, and his physical examination was unremarkable. Initial ECG showed a widespread end-QRS notch with J-point elevation mimicking ST elevation and Q waves in the inferior leads (Figure 1). Cardiac biomarkers were within normal limits on presentation. The urine toxicology screen was positive for tetrahydrocannabinol. The rest of the laboratory findings were within normal limits.

The patient visited the ED a month ago with similar chest pain, and an ECG showed similar findings. At that time, the patient was hemodynamically stable. He underwent cardiac catheterization, revealing coronary arteries free from significant obstruction. During this admission, given the similar nature of the presentation and no changes from the prior ED visit, the patient was monitored over telemetry overnight with no significant events. An echocardiogram revealed a normal LV ejection fraction with a mildly thickened LV wall, grade I diastolic dysfunction, and no pericardial effusion, unchanged from the prior echocardiogram. Serial cardiac biomarkers were unremarkable, and the patient was pain-free. The patient was discharged with no further ischemic risk stratification.

## Discussion

In the time-critical diagnostic dilemma, identifying the subtleties of ECG plays a momentous role in the setting of possible STEMI in patients presenting to the ED with chest pain. The ST elevation in an ECG can be found in several cardiac and extracardiac conditions. Some examples of extracardiac conditions include pancreatitis, acute cholecystitis, drug use (quinidine, procainamide, digoxin, isoproterenol), hyperkalemia, hypothermia, cerebrovascular accidents, etc. [7]. Important cardiac conditions that can present with ST-segment elevation as ER include subtle STEMI and pericarditis [7]. Interpreting subtle STEMI as benign ER can be life-threatening. On the other hand, misinterpretation of ER as subtle STEMI (overcalls) subjects patients to unnecessary medications or invasive procedures. The risks include a 1.8% rate of severe bleeding [8], a 1.2% rate of stroke with thrombolytic therapy [9], and a 0.6% rate of stroke with percutaneous intervention [10]. Thus, it is important to note that, in an acute setting of chest pain, all other conditions, including STEMI, should be carefully excluded to establish the diagnosis of ER.

The term early repolarization has been in use since historic times. The definition of ER, however, varied according to different papers. The 2015 consensus paper on ER pattern by Macfarlane et al. [1] defines ER if all of the following criteria are met: a) end-QRS notch or slur on the downslope of a prominent R wave. If the notch is present, it should lie entirely above the baseline, and if the slur is present, the onset of the slur must also be above the baseline; b) the amplitude at the peak of the notch (J point) is ≥ 0.1 mV in two or consecutive leads excluding V1 to V3; c) the QRS duration should be < 120 milliseconds. A clinical acumen with a detailed history and ECG characterization may be beneficial to differentiate ER from subtle STEMI or acute pericarditis in an acute setting. A younger age at presentation, such as in this patient, might favor early repolarization. The presence of recent viral flu-like illness, positional change in chest pain, pericardial rub, diffuse ST elevation in all ECG leads without reciprocal changes, PR segment depression, and pericardial effusion on echocardiograms indicate acute pericarditis [7]. It should be noted that the presence of convex bowing ST-segment elevation with reciprocal changes and the presence of Q waves should cause concern for myocardial infarction. A comparison with prior ECG findings remains crucial while evaluating patients with chest pain in the ED. In this case report, the initial findings of Q waves in the inferior leads with subtle ST-segment elevation prompted cardiac catheterization in the initial visit, even though the patient was very young. However, upon subsequent presentation with similar chest pain, similar ECG findings, and prior negative invasive studies, the patient underwent conservative management.

Besides acute presentation as chest pain, ER is clinically significant as patients with ER patterns in 12-lead ECG are at higher risk for life-threatening ventricular arrhythmia and sudden cardiac deaths. A study by Tikkanen et al. [3] reported the prognostic significance of ER in a large population group (about 10,800) in which ER in the inferior leads was associated with an increased risk of death from cardiac causes in middle-aged participants. A study by Hasegawa et al. [11] reported the association of ER with an increased risk of atrial fibrillation. However, the risk of sudden cardiac death in asymptomatic patients is very low, and further evaluation is not warranted [4].

In our case report, the patient underwent cardiac catheterization on the initial presentation, demonstrating no obstructive coronary artery disease. Based on the typical nature of chest pain, ECG findings suggestive of Q-wave in the inferior leads, and early repolarization confounding the clinical findings, cardiac catheterization was deemed necessary. However, in a young patient with no significant cardiac risk factors, this might be an overcall. Knowledge of the clinical causes of ST elevation and appropriate risk stratification based on risk factors helps prevent overcalls as well as undercalls in an acute setting.

## Conclusions

Electrocardiographic ER is relatively common in younger populations. It presents with J-point notching in multiple leads, which can sometimes be difficult to distinguish from subtle STEMI or acute pericarditis, especially in time-sensitive decision-making in the ED. A thorough clinical history, review of prior ECGs, and thoughtful risk stratification based on the clinical profile are necessary for an acute setting of chest pain. This helps to minimize overcalls and, at the same time, helps to avoid undercalls while evaluating ED patients with chest pain and subtle J-point elevation.
